# Depth drives microbial assembly while localized tourism selectively enriches bacterial indicator taxa in Lake Fuxian, China

**DOI:** 10.3389/fmicb.2026.1742635

**Published:** 2026-04-10

**Authors:** Jing Chen, Yuwen Xu, Feng Qin, Da-zhong Yan, Hong-Jun Chao, Jing Wu, Yufei Hu

**Affiliations:** 1 College of Life Science and Technology, Wuhan Polytechnic University, Wuhan, China; 2 Institute of Basic Medical Sciences & School of Basic Medicine, Chinese Academy of Medical Sciences & Peking Union Medical College, Beijing, China

**Keywords:** archaeal communities, bacterial communities, Lake Fuxian, tourism-related disturbance, vertical distribution

## Abstract

**Introduction:**

Microbial communities play crucial roles in maintaining lake ecosystem stability. In this study, high throughput 16S rRNA gene sequencing combined with diversity and multivariate statistical analyses was used to investigate how anthropogenic activities, particularly tourism, influence the vertical distribution and composition of bacterial and archaeal communities in Lake Fuxian, a deep oligotrophic freshwater lake in southwestern China.

**Methods:**

A total of 26 bacterial and 3 archaeal phyla were identified, among which Proteobacteria (20.12–32.80%) and Cyanobacteria (17.08–30.39%) dominated the bacterial communities, while Thaumarchaeota (73.29–99.90%) dominated the archaeal ones. Bacterial *α*-diversity was overall higher than that of archaea but showed no significant variations across depths or sites, whereas archaeal diversity varied significantly with depth but not between sites.

**The analysis and results:**

Clustering, NMDS and redundancy analysis (RDA) revealed that depth established the primary ecological scaffolding for both bacterial and archaeal communities. However, tourism drove a localized, taxon-specific restructuring of bacterial communities in the upper layers, while archaeal communities were highly resistant to horizontal spatial disturbances overall. LEfSe analysis showed that *Prochlorotrichaceae* and *Prochlorothrix_PCC-9006* were significantly enriched at the tourism-impacted site L, whereas *Acidobacteria* and *Sphingomonas* were dominant at the deep-water site D. Correlation and RDA analysis indicated that *Cyanobium_PCC-6307* was positively correlated with TN (*p* ≤ 0.01), while the ammonia-oxidizing archaea *Candidatus Nitrosopumilus* and *Candidatus Nitrosoarchaeum* displayed opposite relationships with depth and nutrient parameters.

**Discussion:**

These findings highlight that tourism-related disturbance may alter upper-layer bacterial community composition in deep oligotrophic lakes and provide a critical theoretical basis for evaluating the micro-ecological risks of anthropogenic disturbances, guiding the sustainable tourism development and environmental management of deep plateau lakes.

## 1 Introduction

Lakes are critical freshwater ecosystems that provide essential ecological services, including water storage, nutrient cycling, and habitats for diverse aquatic organisms (Montenegro-Díaz et al., 2025; Sterner et al., 2020). Among them, deep oligotrophic lakes, such as Lake Fuxian in southwestern China, are particularly important due to their high water clarity, ecological sensitivity, and slow nutrient turnover (Zhou et al., 2018; Zhu et al., 2024). However, the expansion of human activities, especially tourism, increasingly threatens these fragile systems by altering nutrient dynamics and disrupting natural habitats (Hadwen et al., 2005; Senetra et al., 2020).

Tourism development around lakes often leads to elevated nutrient inputs, habitat modification, and pollution through runoff, recreational activities, and infrastructure expansion (Li J. et al., 2020; Pang et al., 2022). Such disturbances can accelerate eutrophication, shift water chemistry, and alter ecological interactions, ultimately impacting ecosystem stability (Cai et al., 2024; Vinçon-Leite and Casenave, 2019; Klimaszyk et al., 2020). In this context, microorganisms, particularly bacteria and archaea, serve as both essential drivers and sensitive indicators of ecosystem function, due to their roles in biogeochemical cycling and rapid responses to environmental changes (Tran et al., 2019; Sagova-Mareckova et al., 2020; Zhao et al., 2023). Bacterial communities are generally highly diverse and adaptable, making them particularly sensitive to environmental perturbations such as nutrient enrichment or anthropogenic pollution (Roy et al., 2022). In contrast, archaeal communities are often more stable but display stratified distribution patterns along physical gradients, including depth, temperature, and oxygen availability (Tian et al., 2024).

Lake Fuxian, one of China’s deepest and clearest freshwater lakes, is surrounded by popular scenic areas that attract substantial tourist activity (Li J. et al., 2024). While its pristine waters and biodiversity make it a valuable ecological and recreational resource, increasing tourism and associated infrastructure could threaten its oligotrophic status and microbial ecosystem balance (Cheng et al., 2021; Li Y. et al., 2024). Previous studies have examined microbial vertical distribution in Lake Fuxian (Xing et al., 2020; Xie et al., 2024b; Xie et al., 2024a), but few have addressed how tourism-induced human activity shapes microbial community composition across depths.

In this study, we compared microbial communities between a tourism-impacted site and a relatively undisturbed deep-water site in Lake Fuxian. Our objectives were to (1) evaluate how microbial diversity and composition vary with depth and anthropogenic pressure; (2) determine the differential responses of bacterial and archaeal taxa to environmental gradients; and (3) identify potential microbial indicators of tourism-related disturbance. By integrating vertical stratification data with environmental parameters, this study aims to provide insights into the mechanisms by which human activities influence microbial ecology in deep freshwater lakes and inform sustainable lake management strategies.

## 2 Method

### 2.1 Study area

**Figure 1:**
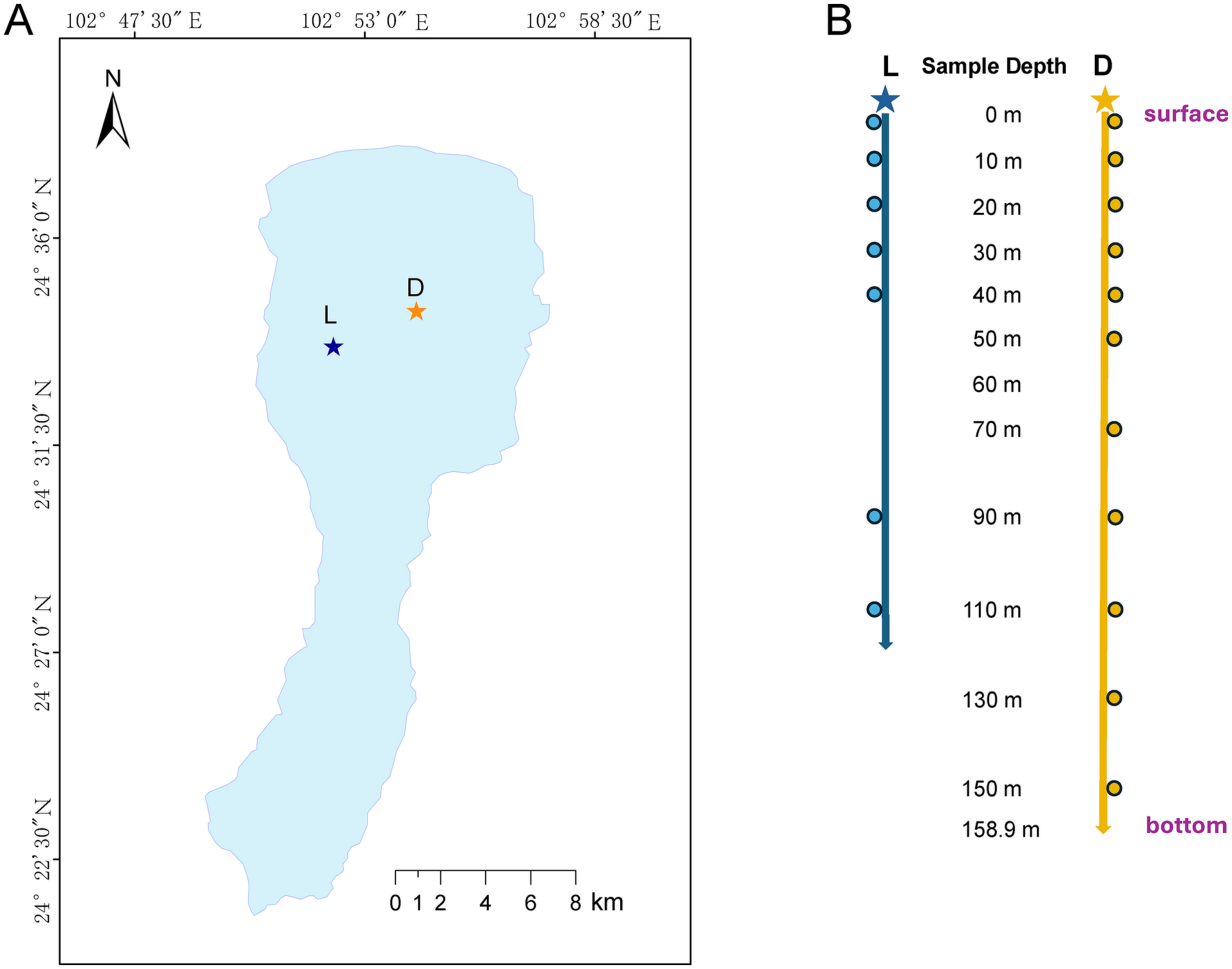
Map of Lake Fuxian and the sampling depths at different sites. **(A)** Sampling site, site in Luchong scenic area (L); the deepest site of Lake Fuxian (D); **(B)** sampling depth for site L and site D.

Lake Fuxian (24°17′–24°37′N, 102°49′–102°57′E) is an oligotrophic plateau lake located at an elevation of 1720 m in Yuxi City, Yunnan Province, Southwest China (Figure 1). Covering a surface area of 212 km^2^, with a maximum depth of 157.8 m, an average depth of 95.2 m, and a total water volume of 18.5 × 10^9^ m^3^, Lake Fuxian is the largest deep freshwater lake in China and serves as a major ecological and tourism resource in the Yuxi region (Li B. et al., 2020).

### 2.2 Sample collection and chemical analysis of water samples

Water samples were collected from two sites in Lake Fuxian: site L (24.56072°N, 102.87093°E) is located in the Luchong Scenic Area, the earliest developed and most densely visited tourist hub around Lake Fuxian, characterized by frequent recreational activities such as boating, sightseeing, and shoreline tourism infrastructure. Consequently, this site experiences higher levels of human disturbance associated with tourism development. The selection of these two contrasting sites allowed us to compare microbial community characteristics between a relatively natural lake environment and an area influenced by tourism-related activities. In contrast, site D (24.57358°N, 102.90400°E), located at the deepest central area of the lake, far from shoreline activities and therefore considered to represent a relatively natural condition with minimal direct anthropogenic disturbance (Figure 1).

Sampling was conducted using a deep-water sampler (2.5 L, 5079A-2500, Sampling Systems Ltd., United Kingdom) at multiple depths ranging from the surface (0–0.5 m) to 150 m, including 10, 20, 30, 40, 50, 70, 90, 110, and 130 m. The specific sampling depths for sites L and D are shown in Figure 1. At each depth, 2 L of water was collected and immediately transported to the laboratory on ice for subsequent chemical and microbial analyses.

Subsequently, 1 L of each water sample was filtered through 0.22 μm membranes (Durapore Membrane Filters, Merck Millipore, United States) for microbial analysis. The filter membranes were fast frozen in liquid nitrogen and stored at −80 °C until DNA extraction. The concentrations of ammonium (NH_4_-N), nitrate (NO_3_-N), total nitrogen (TN) and total phosphorus (TP) were determined following standard analytical protocols (Miner, 2006).

### 2.3 DNA extraction, amplification, and sequencing

Microbial genomic DNA from each water sample was extracted from the filter membranes using the E. Z. N. A.® Water DNA Kit (Omega Bio-Tek, United States) according to the manufacturer’s protocol. DNA concentration and purity were determined with a NanoDrop 2000 spectrophotometer (Thermo Fisher Scientific, United States), and DNA integrity was verified by 1% agarose gel electrophoresis.

The V3–V4 region of the bacterial 16S rRNA gene was amplified using the universal primer pair BacF (5′-ACTCCTACGGGAGGCAGCA-3′) and BacR (5′-GGACTACHVGGGTWTCTAAT-3′), while the V4–V5 region of the archaeal 16S rRNA gene was amplified using primers ArchF (5′-GYGCASCAGKCGMGAAW-3′) and ArchR (5′-GGACTACVSGGGTATCTAAT-3′). Each PCR reaction (20 μL) contained 4 μL of 5 × FastPfu buffer, 2 μL of 2.5 mM dNTPs, 0.8 μL of each primer (5 μM), 0.4 μL of FastPfu polymerase, and approximately 10 ng of DNA template. The amplification conditions were as follows: initial denaturation at 95 °C for 3 min; 30 cycles of denaturation at 95 °C for 30 s, annealing at 55 °C for 30 s, and extension at 72 °C for 30 s; followed by a final extension at 72 °C for 10 min.

The PCR products were separated by 2.0% agarose gel electrophoresis and purified using a Universal DNA Purification Kit (Tiangen Biochemical Technology Co., Ltd., Beijing, China). The purified amplicons from each sample were quantified, pooled in equimolar concentrations, and used to construct sequencing libraries. The libraries were sequenced on an Illumina NovaSeq 6,000 platform (Illumina, San Diego, United States) following the standard protocol of Biomarker Technologies Co., Ltd. (Beijing, China). Raw sequencing reads were generated in FASTQ format and have been deposited in the NCBI Sequence Read Archive (SRA) under accession number PRJNA955892.

### 2.4 Data processing and statistical analysis

Raw sequencing reads were initially trimmed using Trimmomatic (v0.33) to remove low-quality bases. Primer sequences were subsequently identified and removed using Cutadapt (v1.9.1), generating high-quality clean reads. Paired-end reads were merged based on sequence overlaps using USEARCH (v10), and chimeric sequences were detected and removed with UCHIME (v4.2). Amplicon sequence variants (ASVs) were inferred using the UNOISE3 algorithm implemented in USEARCH, providing single-nucleotide resolution of microbial diversity. Taxonomic assignment of ASVs was performed using a Naïve Bayes classifier trained on the SILVA reference database.

Alpha diversity indices were calculated based on the relative abundances of ASVs using Mothur (v1.22.2). Rarefaction curves and boxplots of diversity indices were generated in R using the “vegan” package (function *rarecurve*) to evaluate sampling completeness and variability in community richness. One-way ANOVA was performed in R (package “stats”) with multiple test correction (method = “FDR”), and boxplots of Shannon diversity indices for bacterial and archaeal communities were visualized using the “ggplot2” package (function *geom_boxplot*).

To assess beta diversity and community similarity among samples, hierarchical clustering was conducted in R using the UPGMA (Unweighted Pair Group Method with Arithmetic Mean) algorithm based on the binary-Jaccard distance. Additionally, non-metric multidimensional scaling (NMDS) analysis was performed using the “vegan” package (function *metaMDS*) to visualize dissimilarities in community composition among samples.

Line discriminant analysis (LDA) Effect Size (LEfSe) analysis was taken with python (version 2) to find the bacteria and archaea which were significantly different between different sites. Spearman’s rank correlation coefficients were calculated to explore relationships between environmental variables and microbial taxa. The resulting correlation matrices were visualized as heatmaps using the “pheatmap” package in R, with average linkage clustering applied to group similar variables and taxa. To further elucidate the extent to which environmental factors drive the variation in both bacterial and archaeal community structures at the genus level, Redundancy Analysis (RDA) was performed using the ‘vegan’ package in *R. prior* to the analysis, the genus-level absolute abundance matrix were Hellinger-transformed (function *decostand*) and all environmental variables (Depth, TN, TP, NH₄^+^, NO₃^−^) were standardized (Z-score normalized, function s*cale*). Variance Inflation Factors (VIF) were evaluated (function *vif.cca*) to assess multicollinearity among the environmental drivers. The statistical significance of the overall RDA models, individual axes, and explanatory variables was tested using Monte Carlo permutation tests (function *anova.cca*, by = “margin,” 999 permutations, *p* < 0.05). Finally, the RDA plot was visualized using the “ggplot2” package.

## 3 Results

### 3.1 Alpha diversity analysis

A total of 1,435,628 high-quality bacterial reads (average length 414 bp) and 1,436,813 archaeal reads (average length 412 bp) were obtained after quality filtering. The rarefaction curves based on the Shannon index reached clear plateaus for both bacterial and archaeal communities (Supplementary Figure S1), indicating sufficient sequencing depth.

**Figure 2:**
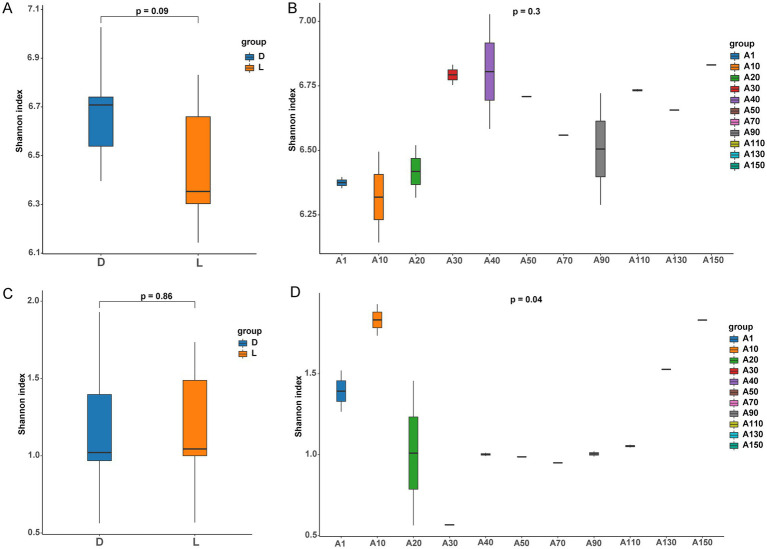
Box plot of Shannon index and one-way ANOVA between different groups of bacteria and archaea. **(A)** Shannon index between site L and site D in bacteria; **(B)** Shannon index among different depths in bacteria; **(C)** Shannon index between site L and site D in archaea; **(D)** Shannon index among different depths in archaea.

The Shannon diversity indices of bacterial and archaeal communities across different sites and depths are shown in Figure 2. For bacteria, the Shannon index at site D was generally higher than that at site L; however, the difference was not statistically significant (Figure 2A; *p* > 0.05). Among depths, bacterial diversity was highest at 40 m, followed by 150 m, but no significant variation was observed among different depths (Figure 2B; *p* > 0.05). In contrast, archaeal communities exhibited much lower Shannon diversity values compared with bacteria. There was no significant difference in archaeal diversity between site D and site L (Figure 2C; *p* > 0.05). However, archaeal diversity varied significantly across depths (Figure 2D; *p* < 0.05), suggesting that vertical stratification had a stronger influence on archaeal community diversity than spatial differences between sites.

### 3.2 Beta diversity analysis

**Figure 3:**
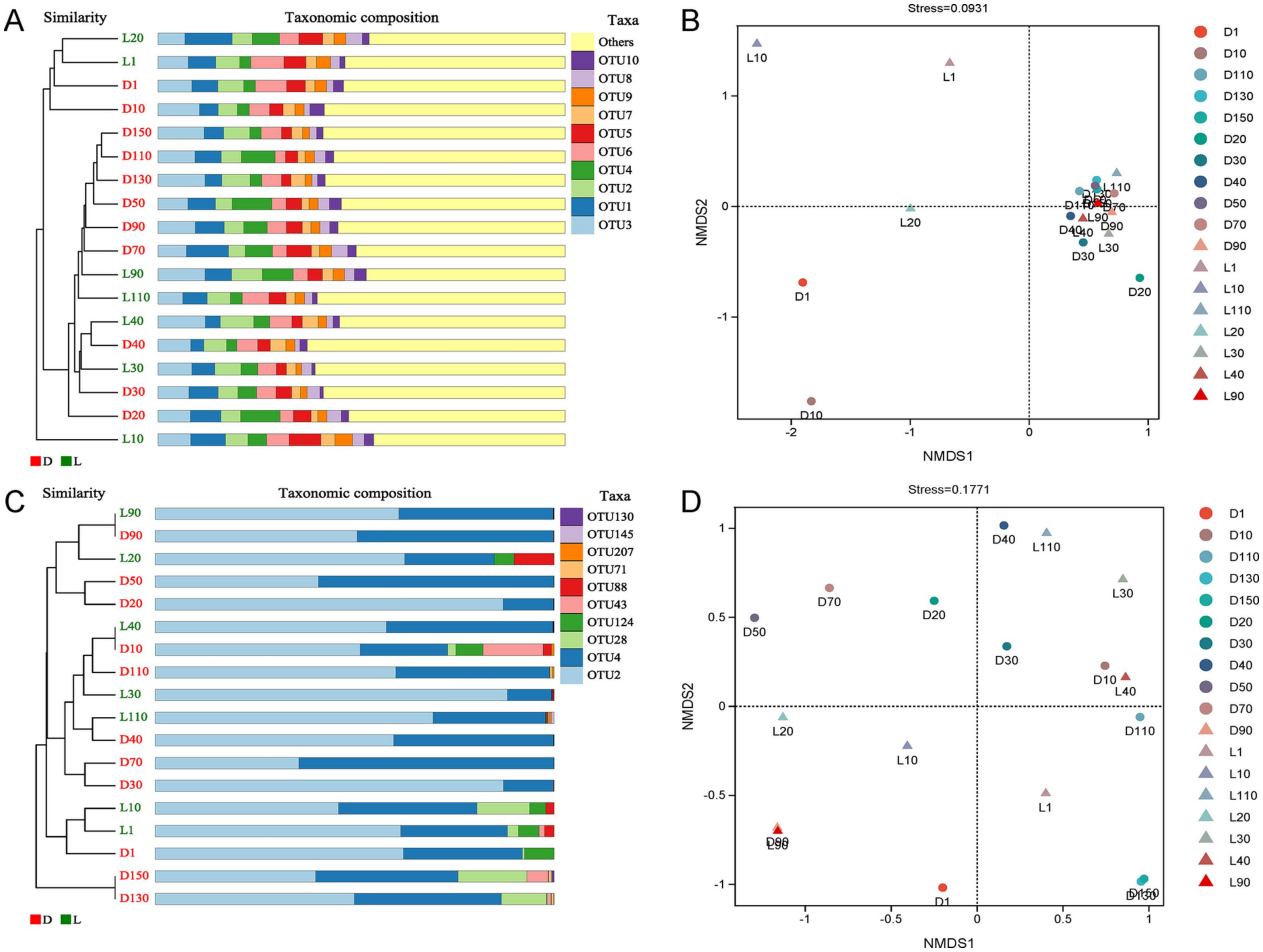
Beta diversity analysis of bacterial and archaeal communities. **(A)** Clustering dendrogram of the bacterial communities; **(B)** NMDS analysis of the bacterial communities; **(C)** Clustering dendrogram of the archaeal communities; **(D)** NMDS analysis of the archaeal communities.

Beta diversity analysis of bacterial and archaeal communities is presented in Figure 3. For bacteria, hierarchical clustering divided the 18 samples into three distinct groups: sample L10 formed an independent cluster separated from all others; samples L20, L1, D1, and D10 grouped together as a second cluster; and the remaining samples constituted a third cluster (Figure 3A). Consistently, NMDS analysis revealed a clear separation of samples L1, L10, L20, D1, and D10—originating from the upper layer of the lake—along the NMDS1 axis. Among these, samples L1, L10, and L20 from site L were further separated from D1 and D10 along the NMDS2 axis (Figure 3B). These results indicate that bacterial community compositions differed markedly between the upper and deep layers, and within the upper layer, the communities at site D were distinct from those at site L.

For archaea, the 18 samples were grouped into four well-defined clusters: D130 and D150 clustered closely together, distinct from all other samples; L1, L10, and D1 formed another cluster; while the remaining samples were distributed into two additional, less structured clusters (Figure 3C). This pattern suggests that archaeal communities at site D, particularly at 130 m and 150 m depths, were similar to each other but distinct from those at shallower depths (1 m and 10 m), which also clustered together. Meanwhile, NMDS analysis revealed that samples of different sites and depths were randomly scattered, implying relatively low beta diversity and weak spatial differentiation in archaeal community structure (Figure 3D).

### 3.3 Bacterial and archaeal compositions of samples

**Figure 4:**
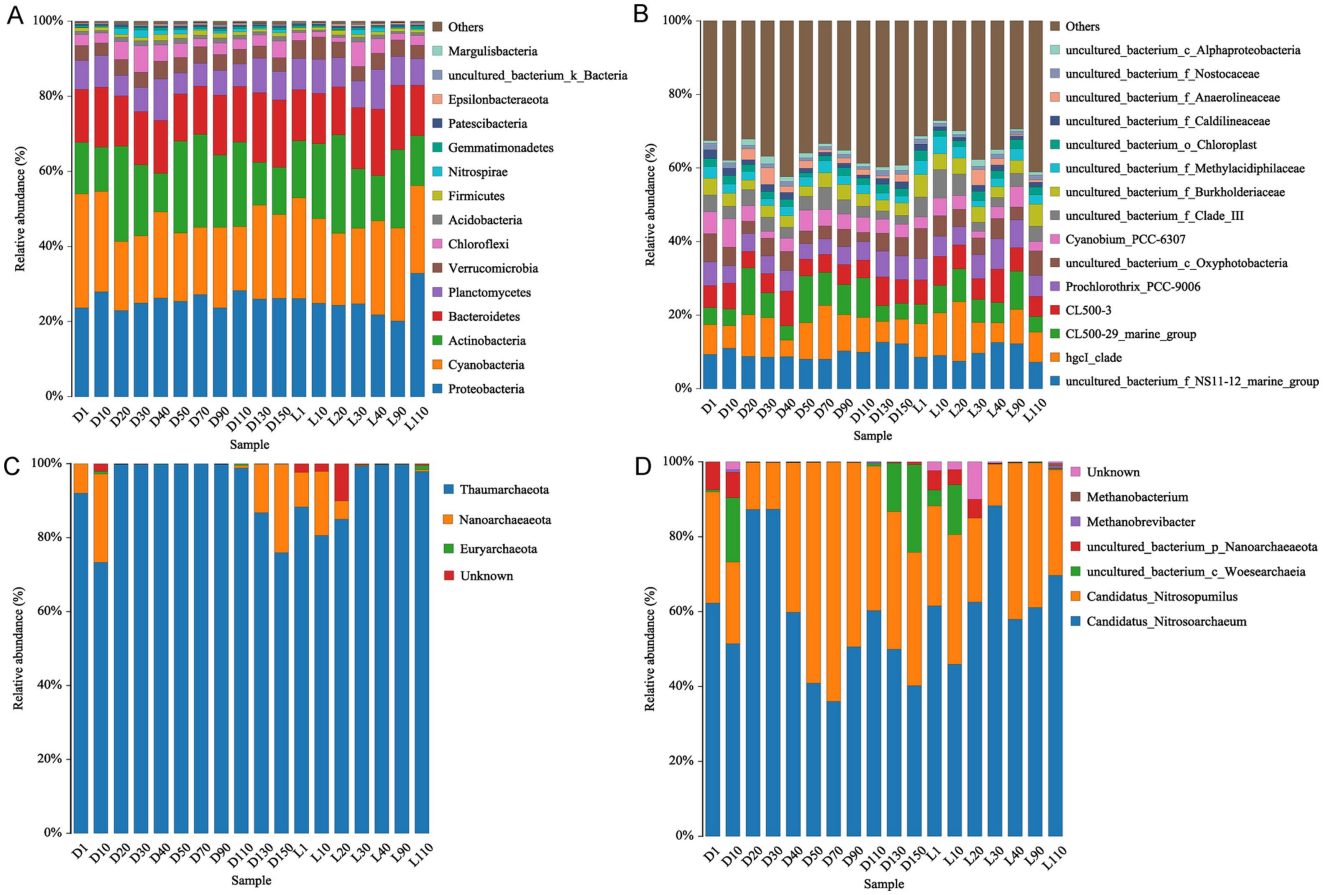
Compositions of bacterial and archaeal communities at different levels: **(A)** Bacterial community compositions at phylum level; **(B)** bacterial community compositions at genus level; **(C)** archaeal community compositions at phylum level; **(D)** archaeal community compositions at genus level.

A total of 26 phyla, 48 classes, 124 orders, 215 families, 344 genera, and 358 species of bacteria were identified across the 18 water samples. The bacteria community compositions on phylum (Figure 4A) and genus (Figure 4B) level were displayed in Figure 4. Proteobacteria (20.12–32.80%) and Cyanobacteria (17.08–30.39%) were the dominant phyla, followed by Actinobacteria, Bacteroidetes, Planctomycetes, Verrucomicrobia and Chloroflex. At the order level, *Sphingobacteriales, Synechococcales* and *Frankiales* were the most abundant (Supplementary Figure S2A). At the genus level, *uncultured_bacterium_f_NS11-12_marine_group* (phylum Bacteroidetes, 7.18–12.63%) and *hgcI_clade* (phylum Actinobacteria, 4.53–16.17%) were the predominant taxa, followed by *CL500-29 marine group, CL500-3 and Prochlorothrix_PCC-9006* (Figure 4B).

For archaea, a total of 3 phyla, 4 classes, 6 genera, and 6 species were detected among the 18 samples. The archaeal community composition at the phylum and genus levels is shown in Figures 4C,D, respectively. Thaumarcheota was the overwhelmingly dominant phylum, with relative abundances ranging from 73.29 to 99.90%, followed by Nanoarchaeaeota and Euryarchaeota (Figure 4C). At the order level, *Nitrosopumilales* was predominant (Supplementary Figure S2B). At the genus level, *Candidatus_Nitrosoarchaeum* (35.98–88.26%) and *Candidatus_Nitrosopumilus* (11.13–63.96%) were the dominant genera (Figure 4D). Both belong to the phylum Thaumarchaeota and are known ammonia-oxidizing archaea (AOA), suggesting that nitrification-related archaea play a central role in the archaeal communities of Lake Fuxian.

### 3.4 Comparisons of bacterial and archaeal communities between different sample sites and depths

**Figure 5:**
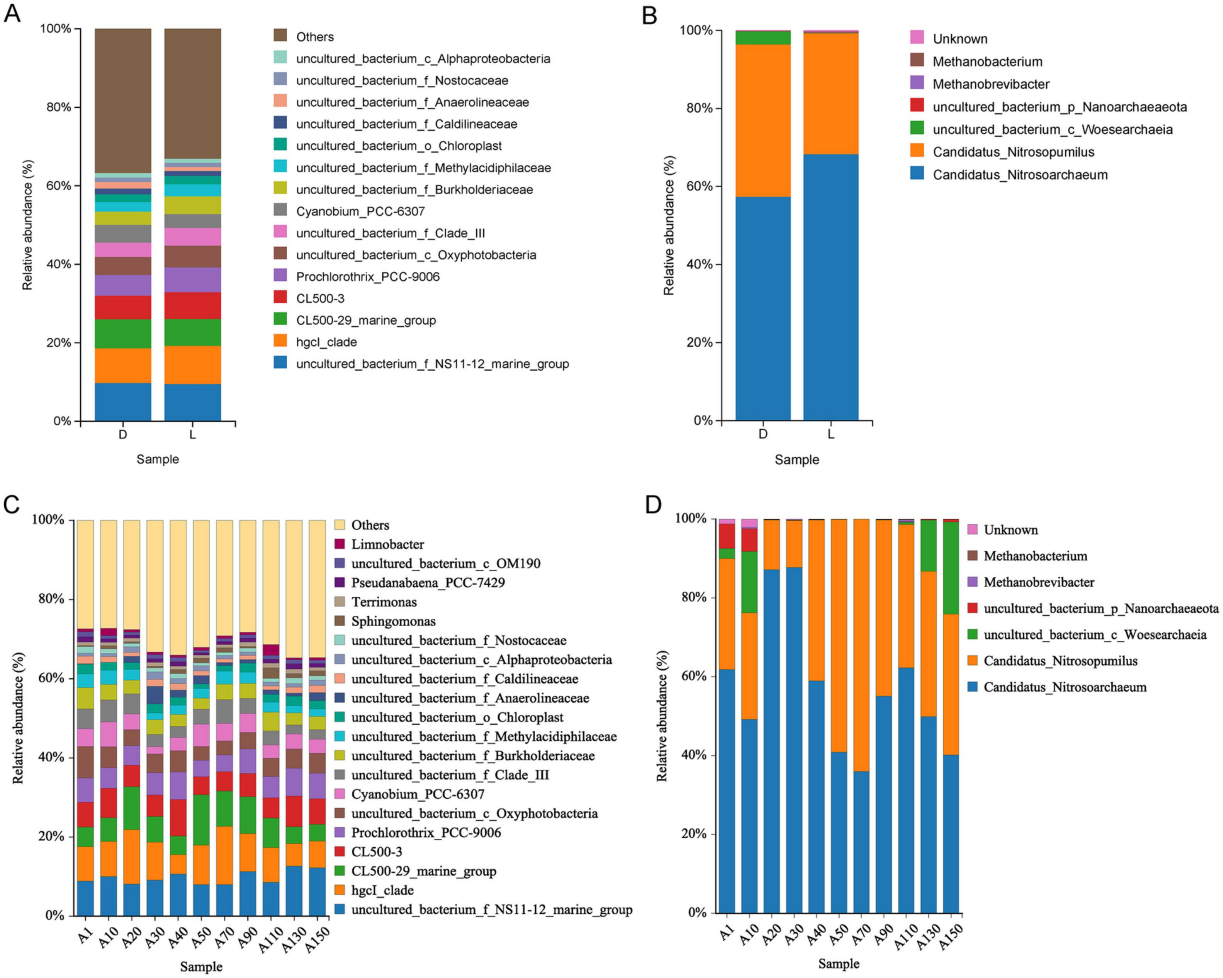
Comparison of bacterial and archaeal communities at genus levels. **(A)** Bacterial community compositions between sites; **(B)** archaeal community compositions between sites; **(C)** bacterial community compositions among depth; **(D)** archaeal community compositions among depth.

To identify bacterial and archaeal taxa that exhibited significant distributional differences between the two sampling sites, we compared the community compositions of site L and site D. Overall, the bacterial community compositions at both sites were largely similar. However, the algal genus *Prochlorothrix_PCC-9006* and the chloroplast-related group *uncultured_bacterium_c_Oxyphotobacteria* were more abundant at site L, whereas *Cyanobium_PCC-6307* was more abundant at site D (Figure 5A).

**Figure 6:**
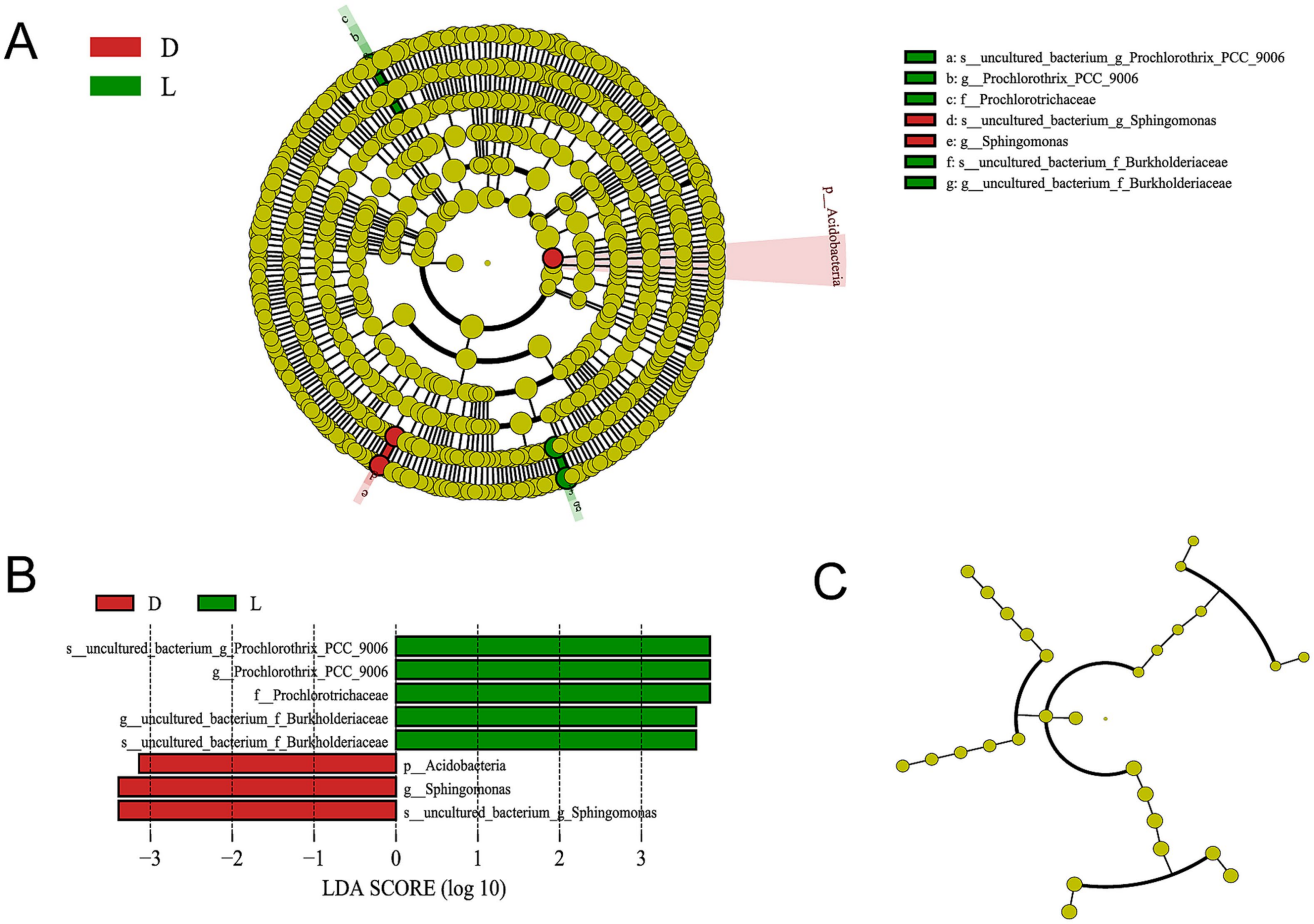
LEfSe analysis of bacteria and archaea between different sites based on LDA scores with a threshold of 3. The significant enriched bacteria or archaea were marked in red (site D) and green (site L). **(A)** Cladogram of bacteria; **(B)** LDA scores of the significantly enriched bacteria; **(C)** Cladogram of archaea.

To further determine the taxa significantly enriched at each site, LEfSe analysis (LDA score > 3.0) was conducted (Figures 6A,B). At site L, the family *Prochlorotrichaceae*, the genera *Prochlorothrix_PCC-9006* and *uncultured_bacterium_f_Burkholderiaceae*, and the species *uncultured_bacterium_g_Prochlorothrix_PCC-9006* and *s_uncultured_bacterium_f_Burkholderiaceae* were significantly enriched, exhibiting high LDA scores. In contrast, the phylum Acidobacteria, genus *Sphingomonas*, and species *uncultured_bacterium_g_Sphingomonas* were significantly enriched at site D (LDA > 3.0; Figures 6A,B). These enriched taxa represent fine-scale biological variations between the two specific spatial locations.

Regarding archaeal communities, *Candidatus_Nitrosoarchaeum* was relatively more abundant at site L, while *Candidatus_Nitrosopumilus* and *uncultured_bacterium_c_Woesearchaeia* were more abundant at site D (Figure 5B). However, LEfSe analysis revealed no archaeal taxa with statistically significant differences between the two sites at the LDA > 3.0 threshold (Figure 6C).

Along the vertical gradient, no clear depth-related patterns were observed for the dominant phototrophic bacterial genera such as *Pseudanabaena_PCC-7429*, *Cyanobium_PCC-6307*, *Prochlorothrix_PCC-9006* and *uncultured_bacterium_c_Oxyphotobacteria* (Figure 5C). Interestingly, the genera *Limnobacter* and *Sphingomonas* showed peak abundances at 110 m depth (sample A110; Figure 5C).

The vertical distribution of archaea exhibited distinct vertical stratification (Figure 5D). The ammonia-oxidizing genus *Candidatus Nitrosopumilus*, detected only in deep-water samples, dominated at 50 m (A50) and 70 m (A70) depths. Meanwhile, the *Candidatus Nitrosoarchaeum* was most abundant at 20 m (A20) and 30 m (A30) depths*. Uncultured_p_Nanoarchaeaeota* occurred primarily in surface waters (A1 and A10), whereas *uncultured_c_Woesearchaeia* was abundant in both surface (A1, A10) and bottom (A130, A150) layers. Methanogenic genera such as *Methanobacterium* and *Methanobrevibacter* were detected at low relative abundances, predominantly in mid-depth samples.

### 3.5 Correlations between environmental parameters and microbial taxa

**Figure 7:**
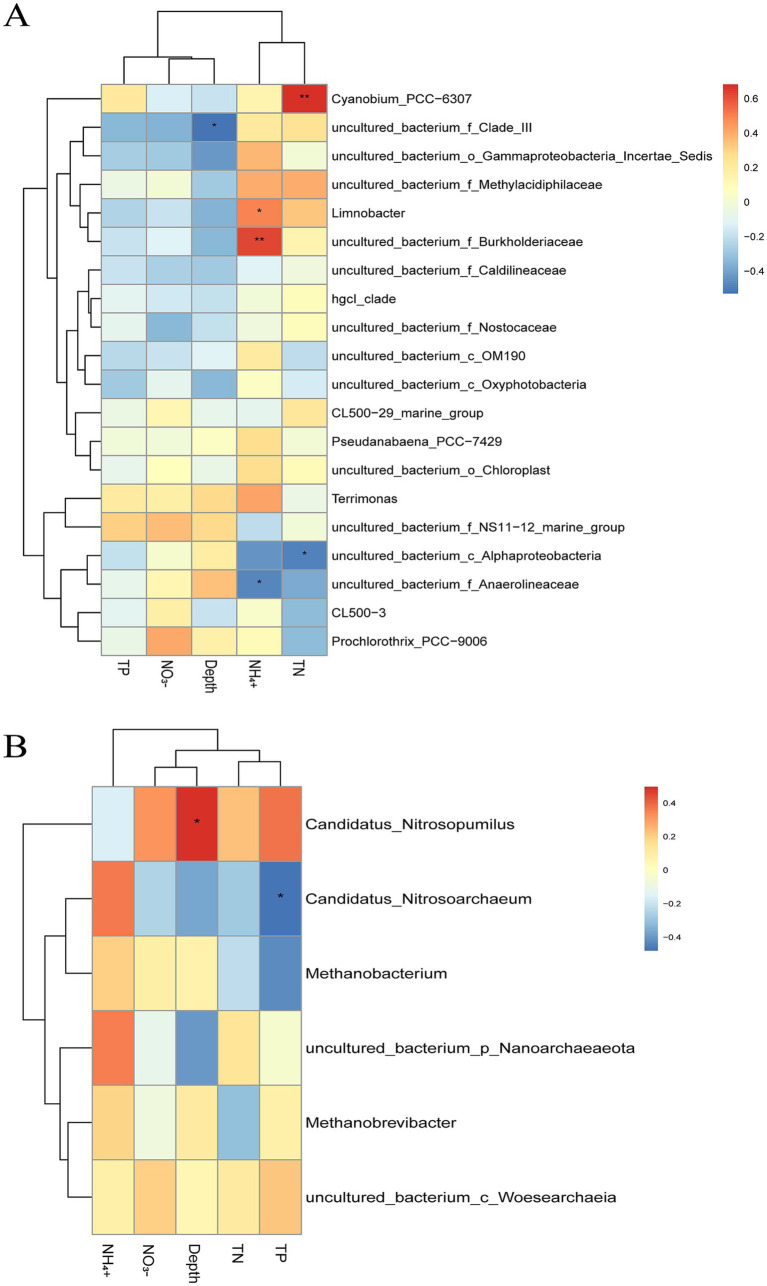
The Spearman *ranking correlation heatmap* between environmental indexes and bacteria **(A)** or archaea **(B)**. R-values representing correlation coefficients are shown in color. *p*-values less than 0.05 are marked by *: *0.01 < *p* ≤ 0.05, **0.001 < *p* ≤ 0.01.

The heatmaps in Figure 7 revealed the relationships between environmental parameters and selected bacterial and archaeal taxa. As shown in Figure 7A, NH₄^+^ was significantly positively correlated with *uncultured_bacterium_f_Burkholderiaceae* (*p* ≤ 0.01) and *Limnobacter* (0.01 < *p* ≤ 0.05), but negatively correlated with *uncultured_bacterium_f_Anaerolineaceae* (0.01 < *p* ≤ 0.05). TN showed a significant positive correlation with *Cyanobium_PCC-6307* (*p* ≤ 0.01), which was previously found to be more abundant at site D than site L (Figure 5A), and a significant negative correlation with *uncultured_bacterium_c_Alphaproteobacteria* (0.01 < *p* ≤ 0.05). Meanwhile, the *uncultured_bacterium_f_Clade_III* was significantly negatively correlated with sample depth (0.01 < *p* ≤ 0.05; Figure 7A).

Among dominant bacterial taxa, *uncultured_bacterium_f_NS11-12_marine_group* (phylum Bacteroidetes) showed positive correlations with TP, NO₃^−^, depth, and TN, but a negative correlation with NH₄^+^. Conversely, *hgcI_clade* (phylum Actinobacteria) exhibited negative correlations with TP, NO₃^−^, and depth, and positive correlations with NH₄^+^ and TN. Meanwhile, the phototrophic genus *Prochlorothrix_PCC-9006*, which was significantly more abundant at site L (Figure 6A), showed positive correlations with NO₃^−^, depth, and NH₄^+^, but negative correlations with TN and TP.

Regarding archaeal communities (Figure 7B), *Candidatus_Nitrosopumilus* showed a significant positive correlation with depth (0.01 < *p* ≤ 0.05), while *Candidatus_Nitrosoarchaeum* was significantly negatively correlated with TP (0.01 < *p* ≤ 0.05). In addition, *Candidatus_Nitrosopumilus* displayed positive correlations with NO₃^−^, TN, and TP, but a negative correlation with NH₄^+^. On the contrary, besides the TP, the *Candidatus_Nitrosoarchaeum* negatively correlated with depth, NO_3_^−^, TN, and positively correlated with NH_4_^+^. Methanogenic genera such as *Methanobacterium* and *Methanobrevibacter* were positively correlated with NH₄^+^, NO₃^−^, and depth, but negatively correlated with TN. Furthermore, *Methanobacterium* showed a negative correlation with TP, whereas *Methanobrevibacter* was positively correlated with TP.

**Figure 8:**
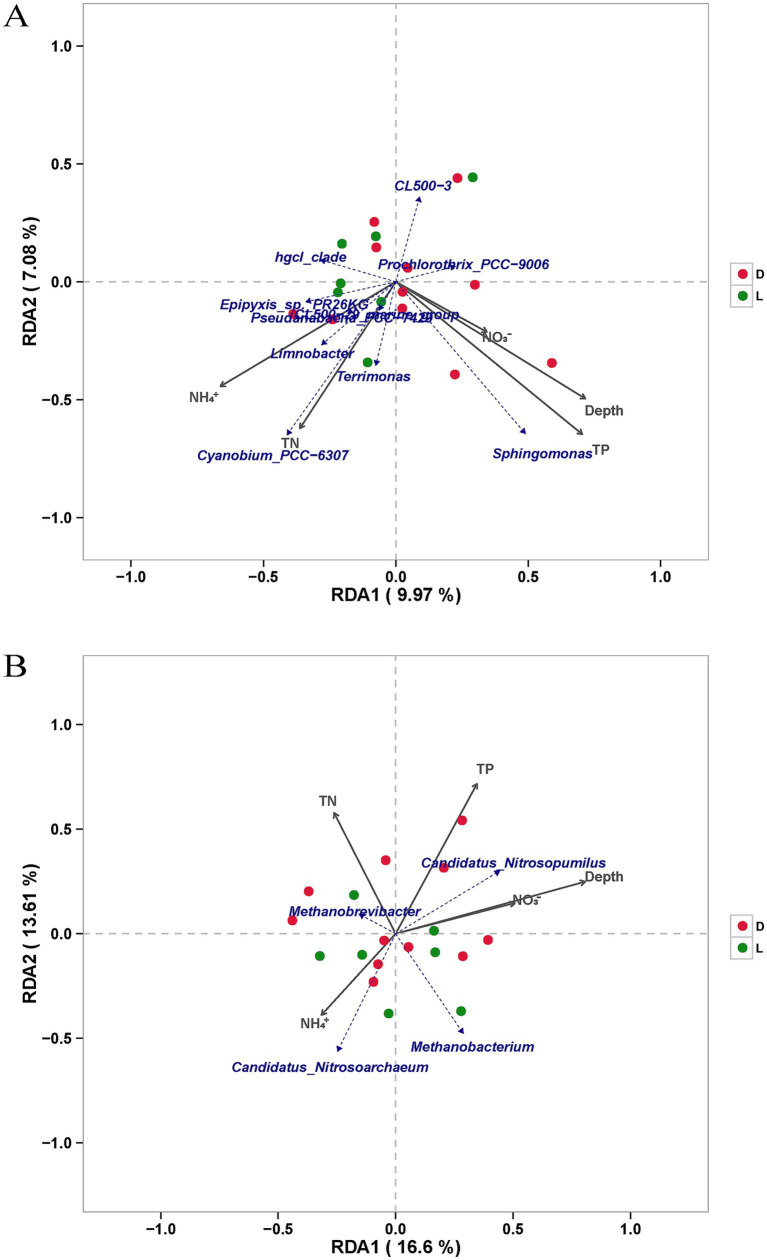
RDA analysis of bacteria **(A)** and archaea **(B)** on genus level. Solid arrows: the environmental index; Dashed arrows: the genus of bacteria **(A)** or archaea **(B)**; Red dots: samples of site D; Green dots: samples of site L.

Consistent with the pairwise Spearman correlations (Figure 7), RDA further elucidated the community-level spatial relationships between the microbial taxa, environmental parameters, and sampling sites (Figure 8). For the bacterial community (Figure 8A), the first two axes explained 17.05% of the total variance, revealing distinct multidimensional distribution patterns between samples from Site D and Site L.

However, to rigorously evaluate these visual patterns, we performed Monte Carlo permutation tests (999 permutations; Supplementary Table S1). The permutation results indicated that individual environmental factors (including tourism-related inputs like NH₄^+^ and TN) did not reach statistical significance in driving the overall bacterial community variance (*p* > 0.05). This suggests that the overall bacterial assemblage possesses a high baseline resilience to horizontal anthropogenic disturbances. Nevertheless, the RDA visually captured localized, taxa-specific associations: the vectors for Depth and TP aligned with deep-water-associated taxa such as *Sphingomonas*, while the TN vector was strictly coupled with *Cyanobium_PCC-6307*, corroborating their preferential distribution at Site D. In contrast, a subset of Site L samples clearly shifted toward the NH₄^+^ vector, which closely aligned with *Limnobacter*. This demonstrates that ammonium enrichment at the tourism-impacted site exerts a localized selective effect on specific indicator taxa, rather than reshaping the entire community structure.

In contrast, the RDA for the archaeal community (Figure 8B), which accounted for 30.21% of the variance, provided robust statistical backing for the striking niche partitioning of ammonia-oxidizing archaea identified in Figure 7B. Crucially, Monte Carlo permutation testing statistically confirmed that natural Depth is a significant primary driver structuring the archaeal community (*F* = 2.70, *p* = 0.019; Supplementary Table S1). *Candidatus_Nitrosopumilus* and samples from Site D were tightly clustered along the Depth, TP, and NO₃^−^ vectors. Conversely, *Candidatus_Nitrosoarchaeum* and samples from Site L shifted completely toward the NH₄^+^ vector, occupying a spatial niche diametrically opposed to the depth gradient. These multivariate results seamlessly integrate with the correlation analyses, statistically validating that the overall archaeal community is strictly anchored by natural vertical stratification, while specific bacterial taxa exhibit localized, fine-scale responses to anthropogenic environmental filters.

## 4 Discussion

### 4.1 Influence of tourism development on bacterial rather than archaeal community diversity

The Shannon index reflects microbial community diversity and ecological stability, with higher values indicating greater diversity and resilience (Shu et al., 2024; Ding and Yu, 2025). In this study, bacterial communities exhibited substantially higher Shannon indices than archaeal communities across all depths, suggesting that bacterial assemblages in Lake Fuxian are more diverse and stable than their archaeal counterparts. This observation aligns with previous findings that bacterial communities generally display stronger adaptability and resilience to environmental fluctuations compared with archaea (Lv et al., 2021). Spatially, although the average bacterial diversity at the natural deep-water site (Site D) was slightly higher than that at the tourism-impacted site (Site L), this difference was not statistically significant. Similarly, archaeal *α*-diversity showed no significant spatial difference between the two sites.

It is important to note that due to the strict ecological protection policies in the Fuxian Lake basin, heavy industry and large-scale agriculture have been strictly prohibited and relocated away from the riparian zones (Li J. et al., 2024). Therefore, within the highly developed Luchong scenic area, the riparian zone is dominated by tourism infrastructure, physically excluding large-scale agricultural practices in its immediate vicinity. Crucially, while overall bacterial α-diversity did not exhibit statistically significant spatial or vertical variation, the subtle localized differences observed in the upper layers of Site L suggest that intensive tourism activities and associated anthropogenic inputs act as a localized disturbance. These specific taxa respond to local anthropogenic nutrients without drastically collapsing the overall diversity baseline of the lake.

In contrast, archaeal diversity exhibited significant vertical differentiation but remained relatively consistent between the two sites, implying that archaeal communities in Lake Fuxian are more strongly structured by natural physicochemical gradients associated with water depth rather than human disturbance.

Together, these results suggest that tourism development introduces localized spatial disturbances that predominantly affect upper-layer bacterial communities, whereas archaeal communities remain highly resistant to anthropogenic impacts and are strictly shaped by natural environmental stratification.

### 4.2 Sampling depth exerts a stronger influence than sampling site on the composition of bacterial and archaeal communities

Given that Lake Fuxian is a deep oligotrophic plateau lake, vertical stratification strongly shapes its physicochemical environment and, consequently, its microbial community structure (Xie et al., 2024b; Xie et al., 2024a). Our results demonstrated that both bacterial and archaeal community compositions were more strongly associated with sampling depth than with sampling site. Hierarchical clustering and NMDS analyses consistently showed that samples collected at similar depths clustered together, regardless of site, whereas samples from different depths within the same site were more distinct (Figures 3A–D). This indicates that vertical gradients, rather than spatial site differences, play a dominant role in structuring microbial assemblages in Lake Fuxian.

For bacterial communities, samples from the upper layers (1–20 m) of both sites were clearly separated from deeper layers, suggesting that the surface and subsurface waters harbor distinct bacterial populations likely shaped by light availability, nutrient concentrations, and oxygen gradients. Notably, the bacterial community at 10 m in the Luchong Scenic Area (L10) exhibited a distinct composition compared to all other samples, as shown by its independent clustering (Figure 3A) and NMDS segregation (Figure 3B). This divergence likely reflects the influence of localized anthropogenic inputs, such as nutrient enrichment and organic matter from tourism activities, which can selectively favor phototrophic or heterotrophic bacterial taxa adapted to more eutrophic conditions.

In contrast, archaeal communities exhibited pronounced stratification with depth rather than spatial variation between sites. The deep-water samples at site D (D130 and D150) formed a distinct cluster (Figure 3C), dominated by ammonia-oxidizing archaea such as *Candidatus Nitrosopumilus*. These taxa are typically adapted to low-light, low-oxygen, and stable nutrient conditions, consistent with the physicochemical environment of deep lake layers (Ngugi et al., 2023; Alfreider et al., 2025; Zheng et al., 2024). Meanwhile, shallower layers (1–10 m) were enriched with *Candidatus Nitrosoarchaeum* and other archaeal groups associated with higher oxygen and nutrient concentrations, forming a separate cluster (Ren and Wang, 2022).

Together, these findings demonstrate that depth-related environmental gradients, such as dissolved oxygen, nutrient availability, and light penetration, are the primary drivers of microbial community composition in Lake Fuxian. While localized anthropogenic disturbance at the Luchong Scenic Area affects surface bacterial assemblages, the overall community structuring of both bacteria and archaea is more strongly governed by vertical stratification than by spatial site variation.

### 4.3 Enrichment of *Cyanobacteria*, *Prochlorotrichaceae*, and *Prochlorothrix_PCC-9006* in the Luchong scenic area of Lake Fuxian

In this study, a total of 26 bacterial phyla were identified, with Proteobacteria (20.12–32.80%) and Cyanobacteria (17.08–30.39%) being the dominant groups, followed by Actinobacteria. These results generally align with previous investigations of Lake Fuxian (Xing et al., 2020; Xie et al., 2024b; Xie et al., 2024a), although the relative abundance of Cyanobacteria in our dataset was notably higher. For instance, Xing et al. (2020) reported Proteobacteria and Actinobacteria as the two most abundant phyla, while Cyanobacteria were comparatively less represented—possibly due to their winter sampling period (December) as opposed to our summer sampling (August). Seasonal shifts, particularly between dry and wet seasons, are known to influence cyanobacterial proliferation in stratified plateau lakes (Wang et al., 2022; Lakshmikandan et al., 2024). The higher relative abundance of Cyanobacteria in our samples thus likely reflects enhanced phototrophic activity and nutrient availability during the warm, stratified season.

Moreover, recent reports have shown a rapid increase in Cyanobacteria abundance in Lake Fuxian, suggesting that the lake may be transitioning toward a mesotrophic or even eutrophic state (Peng et al., 2024; Zhu et al., 2024). While our statistical analyses indicate that the overall bacterial community currently maintains high baseline resilience, this increasing trend raises concerns about the risk of localized cyanobacterial blooms, which could threaten the ecological stability of this oligotrophic plateau lake.

At the genus level, the *uncultured_bacterium_f_NS11-12_marine_group* (7.18–12.63%) and *hgcI_clade* (4.53–16.17%) were dominant taxa, followed by *CL500-29 marine group*, *CL500-3*, and *Prochlorothrix_PCC-9006.* While Xie et al. (2024a,b) reported *CL500-29* marine group and *hgcI_clade* as the dominant genera in Lake Fuxian, our findings revealed a notably higher abundance of *Prochlorothrix_PCC-9006*, a filamentous *Cyanobacterium* belonging to the *Prochlorotrichaceae*. LEfSe analysis further demonstrated that both *Prochlorotrichaceae* and *Prochlorothrix_PCC-9006* were significantly enriched at the Luchong Scenic Area (site L) compared with site D (Figures 6A,B), representing a highly localized, site-specific biological response.

*Prochlorothrix_PCC-9006* is a chlorophyll *b*-containing cyanobacterium capable of oxygenic photosynthesis and is often associated with nutrient-enriched, light-penetrated aquatic environments (Pinevich et al., 2012; Velichko et al., 2014; Strunecký et al., 2023). Its enrichment at site L suggests that localized anthropogenic nutrient input from tourism activities, such as wastewater discharge or organic matter loading from recreational use, may have enhanced the growth of phototrophic bacteria. Consequently, the proliferation of *Prochlorothrix_PCC-9006* and related Cyanobacteria could serve as an early site-specific indicator of local eutrophication and declining water quality in the Luchong Scenic Area. These findings highlight the need for ecological monitoring and stricter management of tourism-derived nutrient inputs to preserve the oligotrophic status of Lake Fuxian.

### 4.4 Nutrient availability and depth govern the distribution of ammonia-oxidizing archaea and specific *Cyanobacteria*

Microbial community composition in aquatic systems is strongly influenced by environmental gradients, particularly nutrient levels and water column stratification (Shilei et al., 2020; Junkins et al., 2022; Yue et al., 2023). In Lake Fuxian, our constrained RDA and permutation tests revealed contrasting community-level responses: while archaeal distributions were significantly driven by natural depth stratification (*p* = 0.019), the overall bacterial community exhibited high baseline resilience to horizontal nutrient gradients (Figures 7, 8; Supplementary Table S1).

Among archaea, two ammonia-oxidizing genera, *Candidatus Nitrosopumilus* and *Candidatus Nitrosoarchaeum*, dominated the community and exhibited striking niche partitioning, which was consistently supported by both analytical approaches. *Candidatus Nitrosopumilus* showed a significant positive correlation with depth and TP (Figure 7B) and strictly aligned with the deep-water samples of Site D in the RDA plot (Figure 8B). This suggests its adaptation to low-light, stable deep-water environments where phosphorus is often regenerated from sediments. In contrast, *Candidatus Nitrosoarchaeum* was negatively correlated with both TP and depth, but positively correlated with NH₄^+^, distinctly pulling the samples from the tourism-impacted Site L toward the NH₄^+^ vector in the RDA space. This indicates a preference for shallower, more oxygenated waters with higher ammonium availability. Similar niche partitioning between these two ammonia-oxidizing archaea (AOA) lineages has been reported in other stratified lakes (Ren and Wang, 2022; Zhao et al., 2024), reflecting their physiological differentiation in response to redox and nutrient gradients. The strong spatial coupling of Site L samples with NH₄^+^ and *Candidatus Nitrosoarchaeum* further suggests that elevated ammonium loading from tourism activities forces a spatial segregation within the nitrifying archaeal communities.

In bacterial communities, although the overall structure remained statistically stable, the multivariate RDA revealed distinct localized distribution patterns between Site D and Site L (Figure 8A). The *Cyanobium_PCC-6307*, a unicellular *Cyanobacterium*, exhibited a significant positive correlation with total nitrogen (TN; Figure 7A) and strictly aligned with the TN vector anchoring the Site D samples in the RDA space. As TN enrichment enhances nitrogen availability, this relationship implies that elevated nitrogen levels may stimulate *Cyanobium_PCC−6,307* proliferation, potentially increasing the risk of cyanobacterial blooms under favorable light and temperature conditions (Chaffin et al., 2018; Esteves-Ferreira et al., 2018; Huisman et al., 2018). Conversely, *Prochlorothrix_PCC-9006*, which was significantly enriched at site L, showed positive correlations with NO₃^−^, NH₄^+^, and depth, but negative correlations with TN and TP. In the RDA plot, it occupied a distinct spatial niche separated from the TN gradient (Figure 8A), suggesting that nitrate and ammonium are its preferred nitrogen sources, while excessive total nitrogen and phosphorus may not directly enhance its growth. Furthermore, the localized enrichment of NH₄^+^ at Site L acted as a specific environmental filter, distinctly pulling a substantial portion of Site L samples toward associated taxa like *Limnobacter*, highlighting a taxa-specific response to anthropogenic inputs.

Collectively, depth-related stratification and nutrient heterogeneity jointly modulate the distribution of key microbial taxa in Lake Fuxian. The distinct responses of AOA and Cyanobacteria to nutrient availability, cross-validated by both pairwise correlations and RDA, reflect the complex interplay between natural nitrogen cycling and anthropogenic nutrient inputs. Importantly, the overall microbial ecosystem exhibits high baseline resilience and remains predominantly anchored by natural vertical stratification. However, continued anthropogenic enrichment, particularly the tourism-derived nitrogen and ammonium loading near the Luchong Scenic Area, induces significant localized, taxa-specific variations, such as the surface enrichment of *Prochlorothrix_PCC-9006*.

From an applied perspective, rather than currently overriding the natural vertical baseline, these fine-scale biological responses serve as sensitive early-warning signals for future ecological shifts. Therefore, we highly recommend incorporating these site-specific indicator taxa into routine lake management and ecological monitoring programs. Furthermore, enforcing strict interception of tourism-derived nitrogenous wastewater (particularly NH₄^+^ and TN) in scenic areas is imperative to prevent localized eutrophication and maintain the lake’s overall oligotrophic stability.

While this study provides high-resolution vertical profiling insights into the microbial assembly of Lake Fuxian, we acknowledge certain limitations in our experimental design that warrant consideration for future research. First, the comparative analysis lacks true large-scale spatial site-level replication. This was largely constrained by the practical challenges of multi-depth sampling, where repeated casts at the exact same location inevitably cause hydrodynamic mixing and disrupt the delicate natural stratification. Second, our investigation was based on a single sampling period during the summer stratification phase (August). Given that microbial communities in plateau lakes are subject to seasonal dynamics, temporal variability across dry and wet seasons could further influence community assembly. Finally, unmeasured confounding factors, such as local hydrological currents or trace metal variations, might also contribute to the observed fine-scale biological variations. Therefore, the site-specific enriched taxa and localized anthropogenic impacts identified here should be interpreted conservatively as snapshot biological responses to fine-scale environmental gradients within this specific ecosystem. Future studies incorporating multi-season tracking and broader spatial replication are recommended to fully validate the universality of these taxa-specific ecological responses.

## 5 Conclusion

This study revealed that natural vertical stratification establishes the primary ecological baseline for both bacterial and archaeal communities in Lake Fuxian. Bacterial communities showed higher diversity and localized spatial variation strictly in the upper layers, indicating greater sensitivity to surface anthropogenic impacts compared to archaea. Tourism-related activities in the Luchong scenic area acted as a localized selective pressure on surface-layer bacterial assemblages, promoting the enrichment of Cyanobacteria, particularly *Prochlorotrichaceae* and *Prochlorothrix_PCC-9006*, which may increase the risk of cyanobacterial blooms. Meanwhile, the positive associations of *Prochlorothrix_PCC-9006* with nitrogenous nutrients (particularly NH₄^+^ and TN) suggest that anthropogenic nitrogen enrichment directly stimulates its localized proliferation, serving as a potential early-warning indicator for cyanobacterial bloom risks. In contrast, archaeal communities were primarily structured by depth and highly resistant to macro-level spatial disturbances. However, tourism-derived nutrient inputs still forced a localized spatial niche segregation between the ammonia-oxidizing archaea, with *Candidatus Nitrosopumilus* and *Candidatus Nitrosoarchaeum* exhibiting opposite correlations with depth and anthropogenic NH₄^+^. Overall, Lake Fuxian’s microbial ecosystem exhibits high baseline resilience, with archaeal communities predominantly driven by natural depth stratification. However, tourism-derived nutrient inputs exert a localized, taxon-specific influence on upper-layer bacterial communities. These localized enrichments of indicator taxa highlight the sensitivity of surface waters, underscoring the imperative for strict wastewater interception in scenic areas. Furthermore, targeting these taxa in future monitoring programs will provide an effective early-warning framework to protect the lake’s overall oligotrophic stability.

## Data Availability

The data presented in this study are publicly available. The data can be found at: https://www.ncbi.nlm.nih.gov, accession PRJNA955892.
