# Supplementary material for: Depth drives microbial assembly while localized tourism selectively enriches bacterial indicator taxa in Lake Fuxian, China
Source: Front Microbiol. 2026 Apr 10;17:1742635. doi: 10.3389/fmicb.2026.1742635 (PMC13107529; doi:10.3389/fmicb.2026.1742635)
Supplement: Supplementary file 1 [file Supplementary_file_1.docx]

**Supplementary information**

**Depth drives microbial assembly while localized tourism selectively enriches bacterial indicator taxa in Lake Fuxian, China**

Jing Chen ^1*^, Yuwen Xu^1^, Feng Qin^1^, Dazhong Yan^1^, Hong-Jun Chao^1^, Jing Wu^1^, Yufei Hu^2*^

1. College of Life Science and Technology, Wuhan Polytechnic University, Wuhan, China.
2. Institute of Basic Medical Sciences & School of Basic Medicine, Chinese Academy of Medical Sciences & Peking Union Medical College, Beijing, China.

^*^ Corresponding authors: Yufei Hu, E-mail: [hyf20@ibms.pumc.edu.cn](mailto:hyf20@ibms.pumc.edu.cn)

Jing Chen, E-mail: chenjingdaisy@126.com

The supplementary materials contain:

Supplementary Figures S1 to S3 marked as Fig. S1, Fig. S2, Fig S3.


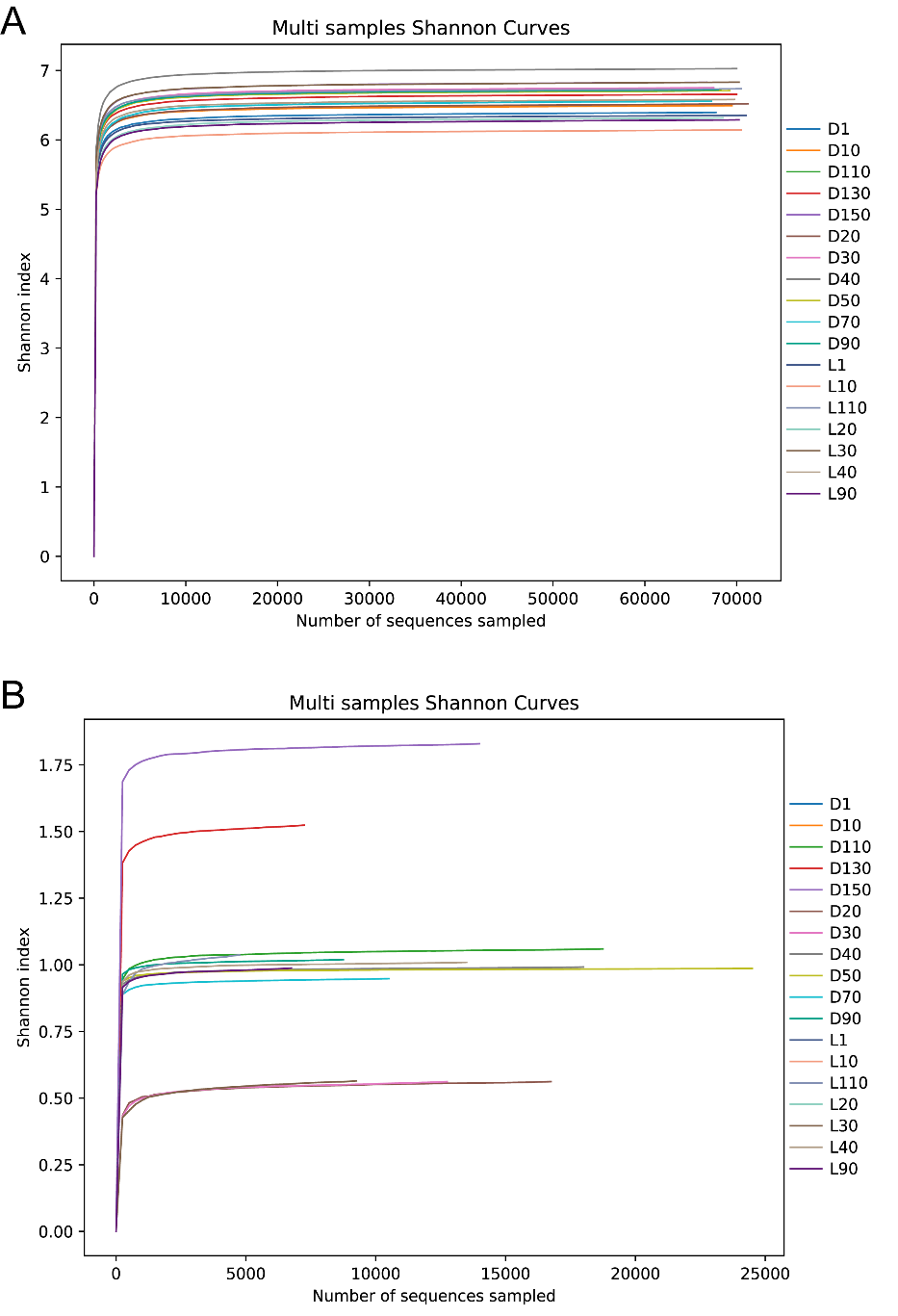


Fig. S1 The rarefaction curves of Shannon index of bacteria (A) and archaea (B).


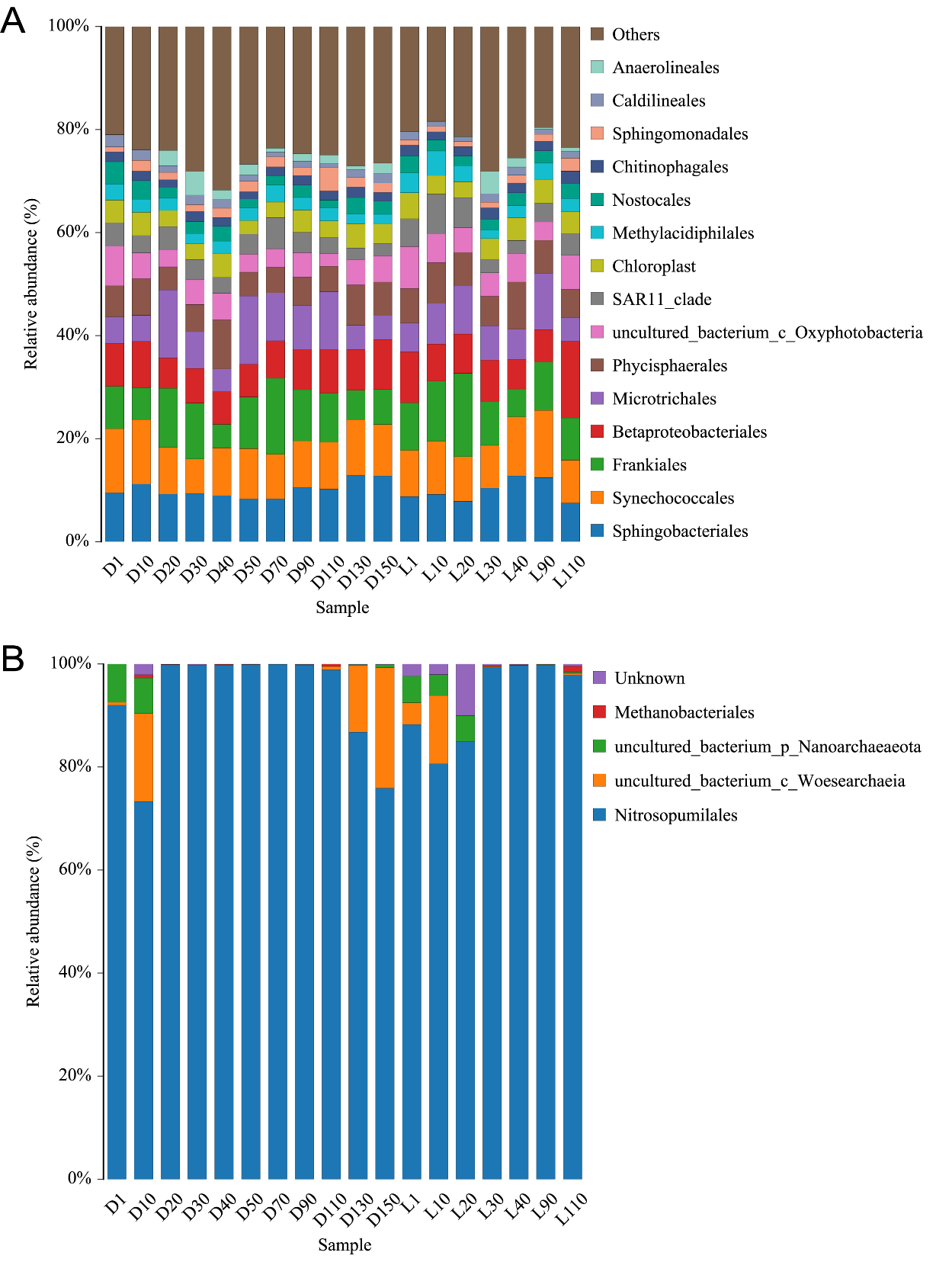


Fig. S2 Compositions of bacterial (A) and archaeal (B) communities at order level.

Table S1 Marginal effects of environmental factors on bacterial and archaeal

communities based on redundancy analysis (RDA) and Monte Carlo permutation tests.

| **Community** | **Environmental Factor** | **Variance** | **F-value** | **P-value** |
| --- | --- | --- | --- | --- |
| **Bacteria** | NH₄⁺ | 0.0044 | 1.25 | 0.250 |
|  | TN | 0.0038 | 1.08 | 0.336 |
|  | TP | 0.0026 | 0.72 | 0.748 |
|  | Depth | 0.0029 | 0.81 | 0.643 |
| **Archaea** | NH₄⁺ | 0.3824 | 1.29 | 0.290 |
|  | TN | 0.4698 | 1.58 | 0.182 |
|  | TP | 0.5776 | 1.94 | 0.096 |
|  | Depth | 0.8039 | 2.70 | **0.019 *** |

*Note:* Statistical significance of the marginal effects was evaluated using Monte Carlo permutations (n = 999). Significant values (*P* < 0.05) are highlighted in bold and with an asterisk. NO₃⁻ was excluded from both RDA models prior to the analysis to mitigate statistical collinearity (Variance Inflation Factor evaluation).
